# Voting Intention and Choices: Are Voters Always Rational and Deliberative?

**DOI:** 10.1371/journal.pone.0148643

**Published:** 2016-02-17

**Authors:** I-Ching Lee, Eva E. Chen, Chia-Hung Tsai, Nai-Shing Yen, Arbee L. P. Chen, Wei-Chieh Lin

**Affiliations:** 1 Department of Psychology, National Chengchi University, Taipei, Taiwan; 2 Research Center for Mind, Brain, and Learning, National Chengchi University, Taipei, Taiwan; 3 Division of Social Science, The Hong Kong University of Science and Technology, Hong Kong S.A.R., China; 4 Election Study Center, National Chengchi University, Taipei, Taiwan; 5 Department of Computer Science and Information Engineering, Asia University, Taichung, Taiwan; 6 Department of Computer Science, National Chengchi University, Taipei, Taiwan; Örebro University, SWEDEN

## Abstract

Human rationality–the ability to behave in order to maximize the achievement of their presumed goals (i.e., their optimal choices)–is the foundation for democracy. Research evidence has suggested that voters may not make decisions after exhaustively processing relevant information; instead, our decision-making capacity may be restricted by our own biases and the environment. In this paper, we investigate the extent to which humans in a democratic society can be rational when making decisions in a serious, complex situation–voting in a local political election. We believe examining human rationality in a political election is important, because a well-functioning democracy rests largely upon the rational choices of individual voters. Previous research has shown that explicit political attitudes predict voting intention and choices (i.e., actual votes) in democratic societies, indicating that people are able to reason comprehensively when making voting decisions. Other work, though, has demonstrated that the attitudes of which we may not be aware, such as our implicit (e.g., subconscious) preferences, can predict voting choices, which may question the well-functioning democracy. In this study, we systematically examined predictors on voting intention and choices in the 2014 mayoral election in Taipei, Taiwan. Results indicate that explicit political party preferences had the largest impact on voting intention and choices. Moreover, implicit political party preferences interacted with explicit political party preferences in accounting for voting intention, and in turn predicted voting choices. Ethnic identity and perceived voting intention of significant others were found to predict voting choices, but not voting intention. In sum, to the comfort of democracy, voters appeared to engage mainly explicit, controlled processes in making their decisions; but findings on ethnic identity and perceived voting intention of significant others may suggest otherwise.

## Introduction

Throughout history, human rationality–the ability to behave in order to maximize the achievement of their presumed goals (i.e., their optimal choices [1])–has fascinated scholars studying human cognition in myriad fields (e.g., economics, sociology, psychology, political sciences). It is also a foundation of the democratic systems. That is, voting is largely considered to be a “deliberate act” [2]: The ability and capacity of individuals to vote with their preferences after deliberation, without having their voting choices (i.e., actual votes) forcibly restricted by external forces, are crucial to the well-being of any democracy [2–3]. In our research, we investigate human rationality in decision-making processes for situations as complicated and consequential as political elections. Understanding how voting decisions in elections may be impacted allows us to evaluate human rationality and the potency of democratic systems.

The degree to which we can make decisions rationally has long been debated. On the one hand, many economists have adopted a utilitarian concept of rationality and have argued for *comprehensive rationality* stance: Human decisions are made in order to maximize the net benefits of the decisions [4]. On the other hand, researchers studying human cognition have found evidence for a *bounded rationality* stance: The ability to make decisions is constrained by environmental restrictions and human capacities [4–6].

However, carefully examining the evidence for the rationality debate has revealed that there are different operational definitions for rationality. For instance, researchers may define optimization differently, depending on the fields in which they investigate rationality. Researchers may rely on mathematical rules (e.g., expected values [7]) or individuals’ subjective views (e.g., preferences in rational choices theory [8], purposes [9], consistency [10]). Although mathematical rules are a common operationalization of rationality, not every choice could be or should be defined in numerical terms.

Therefore, in this study, we define rationality as the ability to behave so that the likelihood of achieving one’s goals is maximized [6, 8]; that is, one behaves in accordance with one’s intentions to achieve a particular goal. If environmental cues and human capacities affect one’s choices outside of one’s intentions, the evidence suggests that one’s rationality is bounded. For example, a person may consider environmental sustainability the most important issue and national security a non-issue when evaluating candidates. As a result, he intends to vote for Candidate A, whose platform includes increased protections for the environment (i.e., *voting intention*). However, on voting day, he votes for Candidate B instead (i.e., *voting behavior*), because the situational cues (e.g., a recent terrorist attack) have prompted his fears regarding national security; he was therefore persuaded by Candidate B’s promises to prioritize national security above all. His vote is not rational, but is bounded by his fears about national security. Thus, we consider an inconsistency between voting intention and voting behavior to be an indication of bounded rationality; consistency between voting intention and voting behavior, on the other hand, would be evidence for comprehensive rationality (e.g., if the person in the above example follows through with his intentions and votes for Candidate A). Independently, factors previously found to affect one’s voting choices (e.g., political party preference) are not necessarily indicators of comprehensive rationality or bounded rationality. If these factors successfully impact both people’s intentions and behavior, they could be considered as support for the comprehensive rationality stance on human cognition. If not, these factors could be considered as evidence for the bounded rationality stance. We aim not only to provide insight into the comprehensive rationality versus bounded rationality debate, but also to evaluate the relevancy of the two types of human rationality in an actual election.

Previous research has found two primary factors in accounting for voting behavior in Taiwan: *political party preference* and *ethnic identity* (see [11] for a review). The Taiwanese political parties can be classified into two categories. The pan-Blue political parties, dominated by the Kuomintang Party (KMT), are largely considered to be more supportive of a closer relationship with the People’s Republic of China [12]. The pan-Green parties, dominated by the Democratic Progressive Party (DPP), are generally thought to be more supportive of Taiwanese independence [12]. Historically, Taipei–the capital of Taiwan–has been a KMT stronghold. However, social events and controversies in the recent years have led to rising public sentiment against the KMT-led government (culminating in the 2014 Sunflower Movement, which saw mass protests in Taipei [13–14]). As a result, during the 2014 Taipei mayoral elections period, support for Wen-Je Ko, an independent candidate perceived to be representing pan-Green interests, grew rapidly; Ko proceeded to successfully challenge the KMT candidate, Sean Lien.

Because elections are often driven by political parties in Taiwan, we separately examined *explicit* and *implicit political party preferences*. Explicit political party preferences for either the pan-Blue or pan-Green camps have been found to be strongly related to voting intention [15] and choices [16], consistent with the comprehensive rationality stance. Explicit political party preferences have also been found to predict voters’ choices elsewhere [17–19]. Accordingly, our first hypothesis was that explicit political party preference should significantly predict voting intention and choices. That is, when respondents favor the DPP over the KMT, they should be likely to express an intention and actually vote for the candidate Ko, and vice versa if they favored the KMT over DPP (expressing an intention and subsequently voting for Lien).

We focused on the more general explicit political party preferences, rather than evaluations of the specific candidates, partisanship, or party identification for three reasons. First, we did not target evaluations of the specific candidates to avoid conceptual conflation with voting intention. Second, we did not target partisanship or party identification because a good proportion of Taiwanese voters (e.g., about 40%) often do not reveal their partisanship [20], especially when their parties become unpopular [21]. Third, less educated Taiwanese people tend not to consider themselves partisans [21]. If the general explicit political party preferences of voters predict both their voting intention and choices, these results support the comprehensive rationality stance.

In addition to explicit political party preference, we examined implicit political party preference. The impact of implicit political party preference on voting intention and choices is more difficult to predict. Galdi and colleagues [22] examined voting intention during an election to determine whether a U.S. military base located in Vicenza, Italy should be expanded. The explicit attitudes regarding the base expansion enlargement best predicted decided respondents’ voting intentions. However, the implicit attitudes regarding the base expansion enlargement best predicted the voting intentions of respondents who stated that they were undecided on the issue. Conversely, when examining voting intention and choices in the 2006 Italian national elections, Roccato and Zogmaister [23] found that although explicit voting intention was the most important predictor for voting choices, respondents’ explicit and implicit political party preferences had separate influences. Specifically, the more respondents favored their preferred political party, explicitly or implicitly, the more likely they intended to vote for the candidate belonging to that party. The researchers also found that when the explicit and implicit political party preferences of the respondents were inconsistent with each other, respondents took more time to make a decision.

Thus, based on the available literature, it is difficult to determine the precise role of implicit political party preference (and the degree of rationality involved) in voting intention and choices. Therefore, we explore the relationship of implicit political party preference with both voting intention and choices. If implicit political party preference can predict both voting intention and voting choices, this evidence would lend support to the comprehensive rationality stance; if implicit political party preference predicted voting choices but not voting intention, then the evidence would provide support to the bounded rationality stance.

In addition to political party preferences, the shift in public opinion before and during the election campaign period in Taipei meant that other factors were also likely to have a sizable impact on voting intention and choices. Thus, we examined two other factors: ethnic identity and *the perceived voting intention of significant others*. Ethnic identity (i.e., whether respondents identify as Taiwanese or Chinese) has been found to predict voting choices in Taiwanese presidential elections, in that voters who identify as Taiwanese are more supportive of a pan-Green candidate while those who identify as Chinese are more supportive of a pan-Blue candidate [24]. Because cross-strait issues should be less prominent in the Taipei mayoral election (compared to a nation-wide presidential election), we expect that if ethnic identity should have an effect, its impact would be in line with the bounded rationality stance. That is, although individuals may not deliberately consider their ethnic identity when voting, they may be more likely to vote for the candidate Ko (the DPP-leaning candidate) if they identify more strongly as Taiwanese.

The fourth and final factor we examined is the impact of people who are significant to an individual (i.e., one’s significant others). Taiwan is a society that emphasizes social relationships [25–26]; thus, significant others may impact respondents’ voting intention and subsequent behavior. There is evidence that discussion with other people can lead to shifts in one’s voting choices (e.g., in the U.S. presidential elections [3, 27]). Therefore, we examined the impact of the perceived voting intention of significant others on voters’ intention and choices. If the perceived voting intention of significant others predicted both voters’ intention and subsequent choices, the results would support the comprehensive rationality stance. Conversely, if the perceived voting intention of significant others predicted only voters’ choices but not intention, the bounded rationality stance would be supported.

To summarize, we examined four predictors that are likely to be key in predicting voting intention and voting choices, focusing on the 2014 Taipei mayoral elections: (a) explicit political party preference, (b) implicit political party preference, (c) ethnic identity, and (d) the perceived voting intention of significant others. To our knowledge, our study is the first to systematically examine human rationality by investigating the impact of these four predictors on both voting intention and choices. The inclusion of all four predictors is crucial because doing so allows us to assess whether voters incorporate different types of information into their decisions. The inclusion of both voting intention and choices allows us to assess whether or not voters’ behavior reflect their intentions, which in turn allows us to evaluate the rationality of these individuals’ cognitive processes. If all four predictors were associated with voting intention and choices, the results would suggest that voters are capable of marshalling all relevant information to make their decisions before and during the voting period. However, if the predictors were associated with voter choices but not intention, it is possible that voters may experience some cognitive limitations when considering the information presented to them before they vote, thus casting doubt on our capability for comprehensive rationality.

## Materials and Method

### Ethic Approval

This research was supported by a grant (MOS 103-2221-E-004-007-MY3) to one of the authors (A. L. P. C.) and by financial assistance from the Research Center for Mind, Brain, and Learning, National Chengchi University. The research was approved by the Research Ethics Committee, National Taiwan University (NTU-REC No. 201402EM023). Participants provided written informed consent before they began the study.

### Participants

We targeted residents from all twelve districts in Taipei, Taiwan. We advertised our study using various social media platforms (i.e., Facebook and the Bulletin Board System, platforms that are popular in Taiwan) and through personal connections. In total, 124 respondents (64 males) were recruited. Respondents were eligible voters, and 86.3% were young adults (i.e., adults younger than 40 years of age). Most of the respondents (40.3%) did not indicate any party identification; 38.7% identified with the DPP party; and 19.4% identified with the KMT party. The majority of the respondents (71.0%) had voted in the 2012 presidential election, the last major election in Taiwan.

### Procedure and materials

Approximately one month before the 2014 mayoral elections in Taipei, respondents were invited by phone to complete a survey and an implicit association test (IAT) at a public university in Taipei. The survey (see items in Table 1) asked participants to answer questions on their party identification, explicit political party preference, ethnic identity, and voting intention. Participants also had to report the perceived voting intention for their significant others. To provide a parallel comparison with implicit political party preference (as measured by the IAT), explicit political party preference was calculated by contrasting the respondents’ preference for the DPP over the KMT; that is, the higher the survey scores, the more the respondents preferred the DPP over the KMT. Voting intention and perceived voting intention of significant others were calculated using the same rationale. Voting intention was measured by two items, one item for their intended candidate and one item for the certainty of such a decision. Intended candidate was coded as follows: 1 for the candidate Ko, -1 for the candidate Lien, and 0 for all other candidates. The strength of voting intention was calculated by multiplying the intended choice with the degree of certainty. Similarly, the perceived voting intention of significant others was estimated by multiplying the perceived significant other’s intended candidate choice (coded the same way as for the participant’s intended candidate) with the perceived certainty of the significant other. Higher scores indicate a preference for Ko over Lien, as well as stronger certainty.

**Table 1:** Measurements and reliabilities in the survey (translated from Chinese).

Constructs	Example items
**Political party ID (2 items)**	1. Currently, there are the following major parties in our country: the Kuomintang, the Democratic People’s Party, the People First Party, the New Party, and the Taiwan Solidarity Union Party. Which party are you inclined to support?
	2. To what degree are you inclined to support your chosen party?
**Explicit political party preference (2 items)**^a^^,^^b^	If 0 represents “strongly dislike” and 10 represents “strongly like,” how would you score the two main national parties?
	1. KMT: ______
	2. DPP: _____
**Ethnic ID (one item)**^c^	In our society, some people identify themselves as Chinese, and some people identify themselves as Taiwanese. How would you identify yourself?
**Voting intention (2 items)**^a^	1. If you will vote in the upcoming elections, which mayoral candidate are you more likely to vote for?
	2. How sure are you about your voting intent?
**Perceived voting intention of significant others (2 items)**^a^	1. Please think about a close family member or friend who has the most impact on you in terms of politics. Which mayoral candidate is the person likely to vote for?
	2. How sure is your family member or friend about their voting intent?

^a^Each item taps different aspects of the construct; thus, no reliabilities were calculated.

^b^Explicit political party preference was calculated by contrasting the respondents’ preference for the DPP over the KMT.

^c^Coded 3 for Taiwanese only, 2.5 for Taiwanese priority, 2 for equally half, 1.5 for Chinese priority, and 1 for Chinese only.

Following the survey, respondents took a political party preferences IAT (see S1 Appendix for a detailed description of how the test was developed). The IAT tapped respondents’ implicit associations of valence with the main political parties in Taiwan, the KMT and DPP, by measuring how positive and negative words may reduce or prolong the reaction time of stimuli representing either party. We calculated the resulting D-scores so that higher D-scores indicated a stronger preference for the DPP (i.e., DPP = good). The order of the blocks (i.e., Blocks 3 and 5; see S1 Appendix) within the IAT did not affect respondents’ implicit political party preference scores (*p* = .88).

One week after the conclusion of the Taipei mayoral elections, respondents were contacted again by phone to report their actual voting choice. Responses were coded the same way as in intended candidate: 1 for the candidate Ko, -1 for the candidate Lien, and 0 for all other candidates.

## Results

### Descriptive analysis

On average, respondents explicitly stated that they favored the DPP over the KMT (*M*_D_ = 1.28, *SD* = 4.02), *t*(122) = 3.52, *p* = .001, but they showed no implicit preference for either party (*M* = 0.01, *p* = .82). The explicit political party preferences were consistent with respondents’ party identity. Participants who identified with the DPP showed the most explicit preference toward the DPP over KMT (*M* = 4.38, on a scale of -10 to 10). By contrast, participants who identified with the KMT and participants who did not identify with a party showed significantly less explicit preference for the DPP (*M* = -4.08 and *M* = 0.88, respectively), all pairwise contrasts at *p*s < .01. Furthermore, implicit political party preferences were also consistent with respondents’ party identity, with the DPP identifiers showing the strongest implicit preference toward the DPP (*M* = 0.29), no-party identifiers showing little preference for either party (*M* = 0.02), and KMT identifiers showing a preference for the KMT (*M* = -0.58), all pairwise contrasts at *p*s < .01.

The majority of the respondents identified as Taiwanese only (72.7%). On a scale of 1 (Chinese only) to 3 (Taiwanese only), respondents scored *M* = 2.60, *SD* = 0.67. Most of the respondents (83.6%) indicated that they would vote in the mayoral election, and 69.4% indicated that they would vote for the candidate Ko. Participants were very certain of their decision before the election (on a scale from 0 to10, with 0 indicating strong uncertainty and 10 indicating strong certainty; *M* = 8.22, *SD* = 2.69). Over half (56.4%) also reported that their significant other would vote for the candidate Ko, and that these significant others were also very certain of their choices (*M* = 8.65, *SD* = 2.25).

To examine the degree to which the four predictors–explicit political party preference, implicit political party preference, ethnic identity, and perceived voting intention of significant others–can predict voting intention and choices, we first ran hierarchical regression analyses on voting intention, as well as on choices, respectively. In the first step, we entered respondent gender and education level (with a STEPWISE method). In the second step, we entered the explicit and implicit political party preference scores (with an ENTER method). Finally, we entered ethnic identity, perceived voting intention of significant others, as well as the interaction of the explicit and implicit political party preferences (with a STEPWISE method).

### Analyses for voting intention

**Fig 1:**
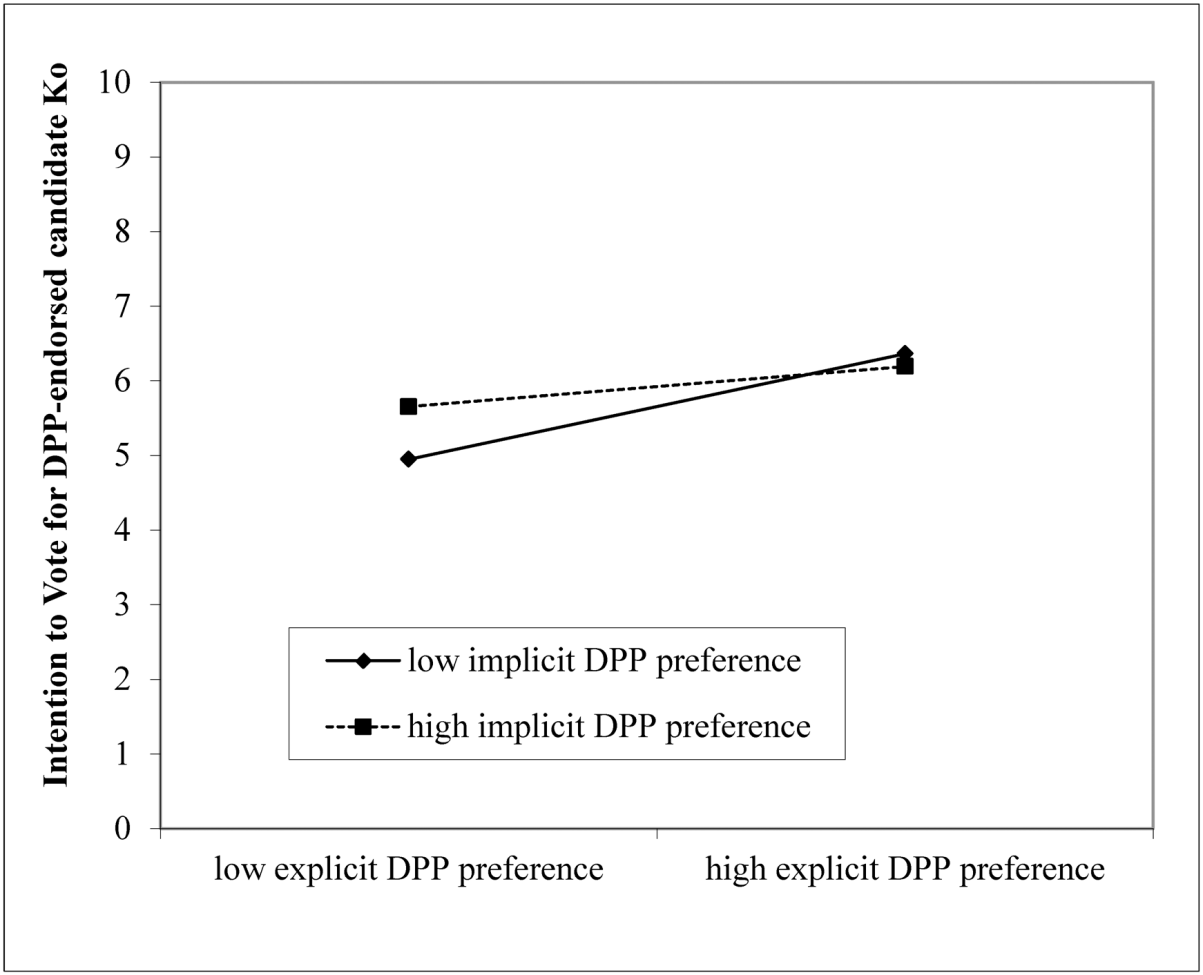
Respondents’ explicit and implicit political party preferences (DPP over KMT) in predicting voting intention. Solid diamond: low implicit DPP preference. Solid square: high implicit DPP preference.

Respondents’ explicit political party preferences predicted their voting intention, standardized *B* = 0.49, *p* < .001, suggesting that the more respondents favored the DPP over the KMT, the more likely they expressed an intention to vote for the candidate Ko. There was also a significant interaction between explicit and the implicit political party preferences, *B* = -0.22, *p* = .001. As seen in Fig 1, there was a steeper slope of respondents with low implicit DPP preference compared to those with high implicit DPP preference. Respondents who had consistently both low explicit and implicit DPP preferences were least likely to express an intention to vote for the candidate Ko. Respondents who had high explicit DPP preference were most likely to express an intention to vote for the candidate Ko, regardless of their implicit DPP preferences. The perceived voting intention of significant others, *B* = 0.15, *p* = .047, and implicit DPP preference, *B* = 0.13, *p* = .07, also predicted respondents’ intention to vote. The more respondents perceived significant others’ intent to vote for the candidate Ko, as well as the more respondents preferred DPP implicitly, the more likely (marginally for implicit preference) they expressed an intention to vote for candidate Ko. No other variables were significant, and the model accounted for 54.8% of the variance.

### Analyses for voting choice

**Fig 2:**
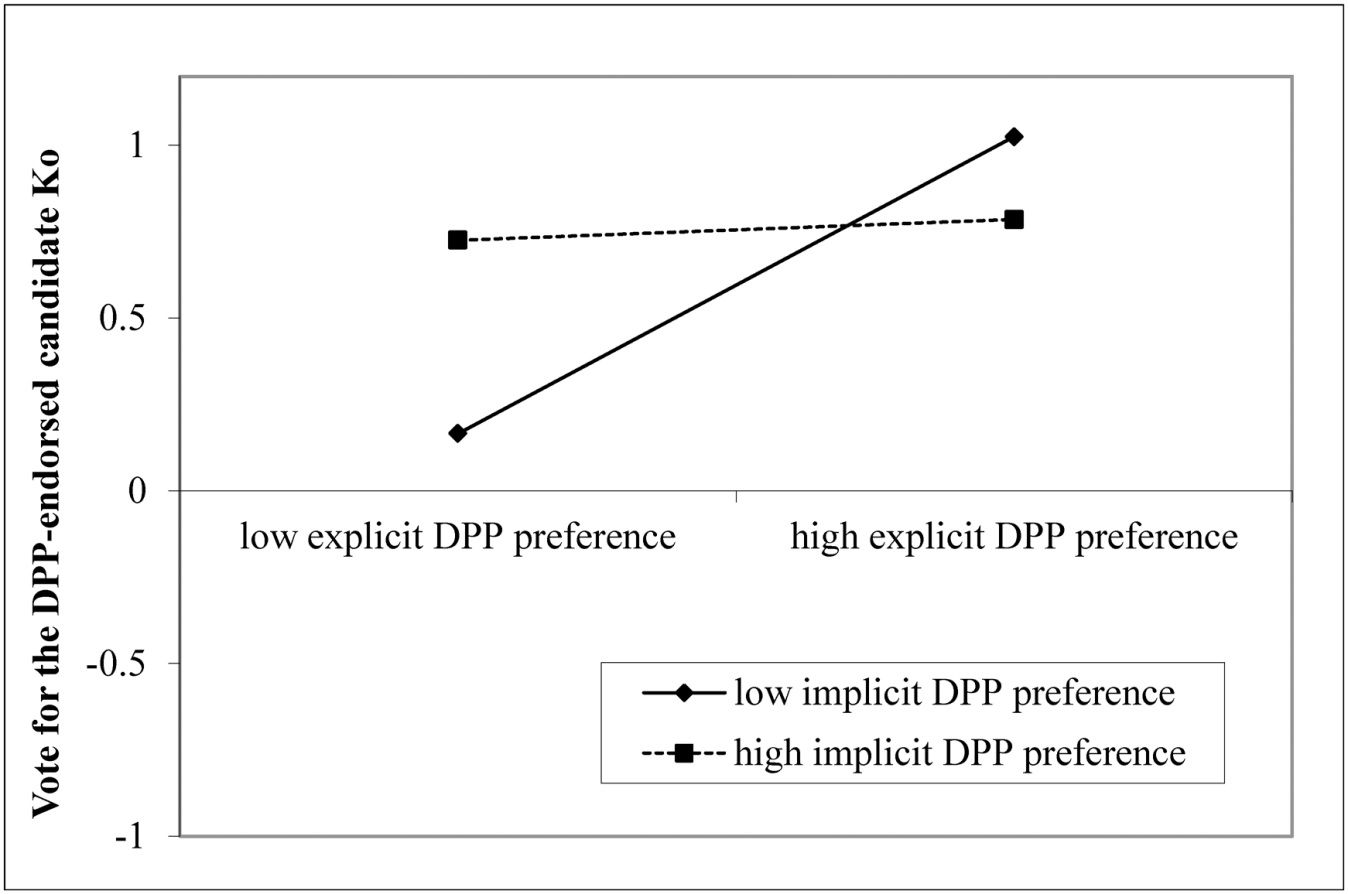
Respondents’ explicit and implicit political party preferences (DPP over KMT) in predicting voting behavior. Solid diamond: low implicit DPP preference. Solid square: high implicit DPP preference.

The explicit political party preference of respondents, *B* = 0.22, *p* = .028, and an interaction between explicit and implicit political party preferences, *B* = -0.21, *p* = .01, significantly predicted their voting choices (see Fig 2), replicating the findings in the analyses for voting intention. Ethnic identity, *B* = 0.23, *p* = .007, and the perceived voting intention of significant others, *B* = 0.19, *p* = .031, were also found to be robust predictors. The interpretations of the effects of explicit political party preferences, the interaction between explicit and implicit political party preferences, and the perceived voting intention of significant others on voting choices were identical to those on voting intention. Respondents who identified themselves as Taiwanese were more likely to vote for the candidate Ko compared to respondents who identified themselves as Chinese. No other variables were significant, and the model accounted for 39.0% of the variance.

### Path model analysis: putting pieces together

**Fig 3:**
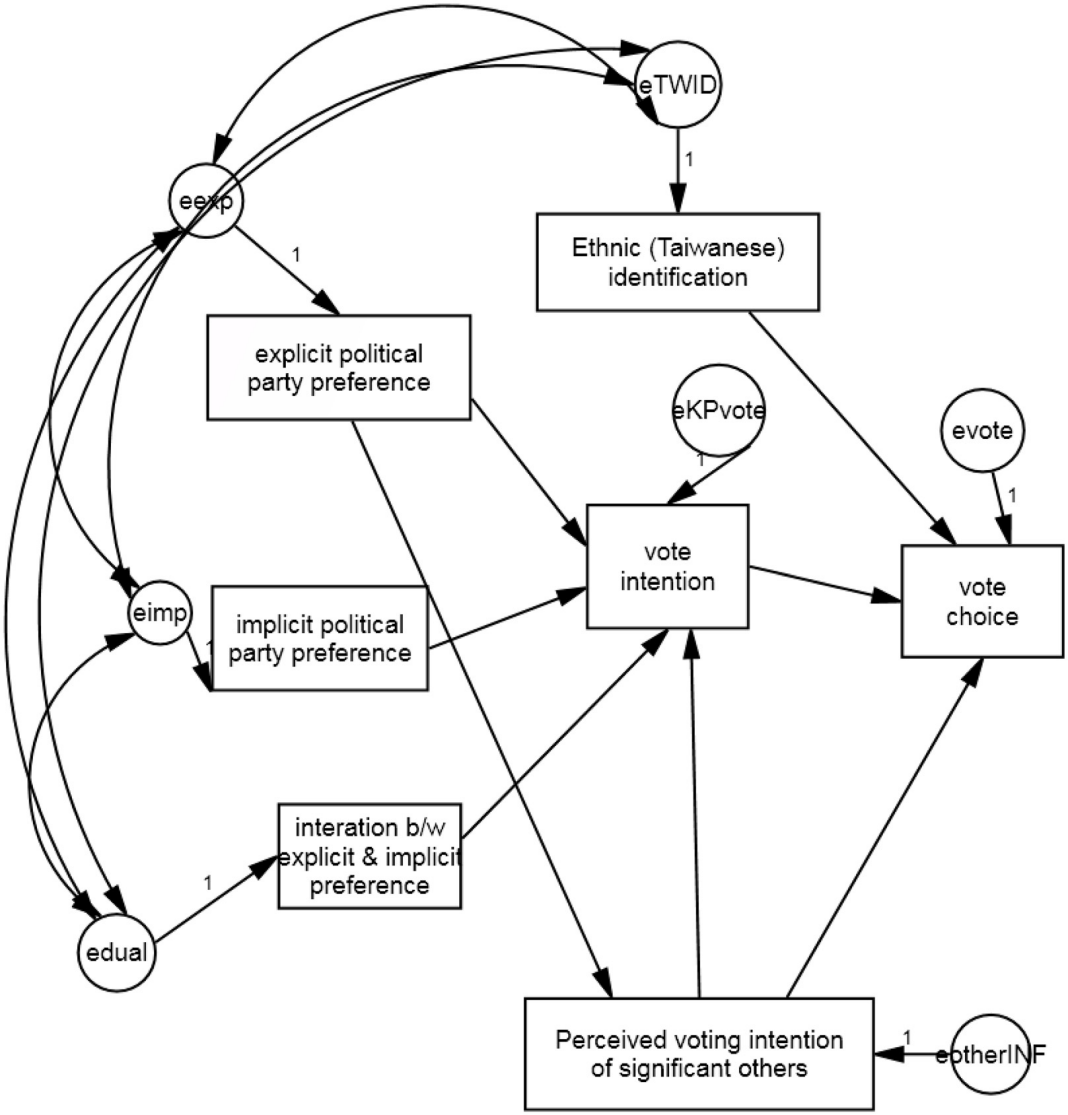
A path model in voting intention and choice.

We conducted a path model analysis with a bootstrapping method (see Fig 3 and the correlation matrix in S2 Appendix). Based on the findings in the regression analyses, explicit political party preferences, implicit political party preferences, the interactions between the two types of party preferences, and the perceived voting intention of significant others were proposed to predict voting intention and choices, respectively. Ethnic identity was proposed to predict voting choices. Because ethnic identity and different levels of political party preferences were often intertwined in political discourse, the error terms of ethnic identity, explicit political party preferences, implicit political party preferences and the interaction term of the two kinds of political party preferences were covaried. Lastly, respondents’ explicit political party preference may affect with whom they would like to be associated. Thus, a path from explicit political party preference to the perceived voting intention of significant others was drawn. When fitting the model with the participants’ responses, the model has a good fit, χ^2^(7) = 8.82, *p* = .27; CFI = 0.99, RMSEA = 0.046. The paths are listed in Table 2.

**Table 2:** Paths in the model predicting voting intention and choice: Standardized coefficients.

Paths	Estimate
Predicting voting intention	
Explicit political party preference → Voting intention	.49***
Implicit political party preference → Voting intention	.10
Interaction b/w explicit & implicit preferences → Voting intention	-.18**
Perceived voting intention of significant others→ Voting intention	.11
Predicting vote choice	
Voting intention → vote choice	.37***
Ethnic (Taiwanese) identity → Vote choice	.24**
Perceived voting intention of significant others→ Vote choice	.18*
Other paths	
Ethnic (Taiwanese) identity ↔ Explicit political party preference	.48***
Ethnic (Taiwanese) identity ↔ Implicit political party preference	.37***
Ethnic (Taiwanese) identity ↔ Interaction b/w explicit & implicit preferences	-.31***
Explicit political party preference ↔ Implicit political party preference	.47***
Explicit political party preference ↔ B/W explicit & implicit preferences	-.32***
Implicit political party preference ↔ B/W explicit & implicit preferences	-.17+
Explicit political party preference → Perceived voting intention of significant others	.50***

***: *p* < .001,

**: *p* < .01,

*: *p* < .05,

^+^: *p* = .06.

Consistent with the findings from hierarchical regression modeling, the explicit political party preferences of the respondents and the interaction between explicit and implicit political party preferences predicted voting intention. Once voting intention was taken into account, respondents’ explicit political party preference and the interaction term were no longer significant predictors for voting choices. Thus, voting intention served as the complete mediator between (a) the explicit political party preferences to voting choices, Sobel Z = 3.67, *p* = .0002, and (b) between the interaction of implicit and explicit political party preferences to voting choices, Sobel Z = -2.43, *p* = .015. Lastly, the associations of ethnic identity and perceived voting intention of significant others on voting choices remained significant after controlling for voting intention.

### Supplementary analysis

**Fig 4:**
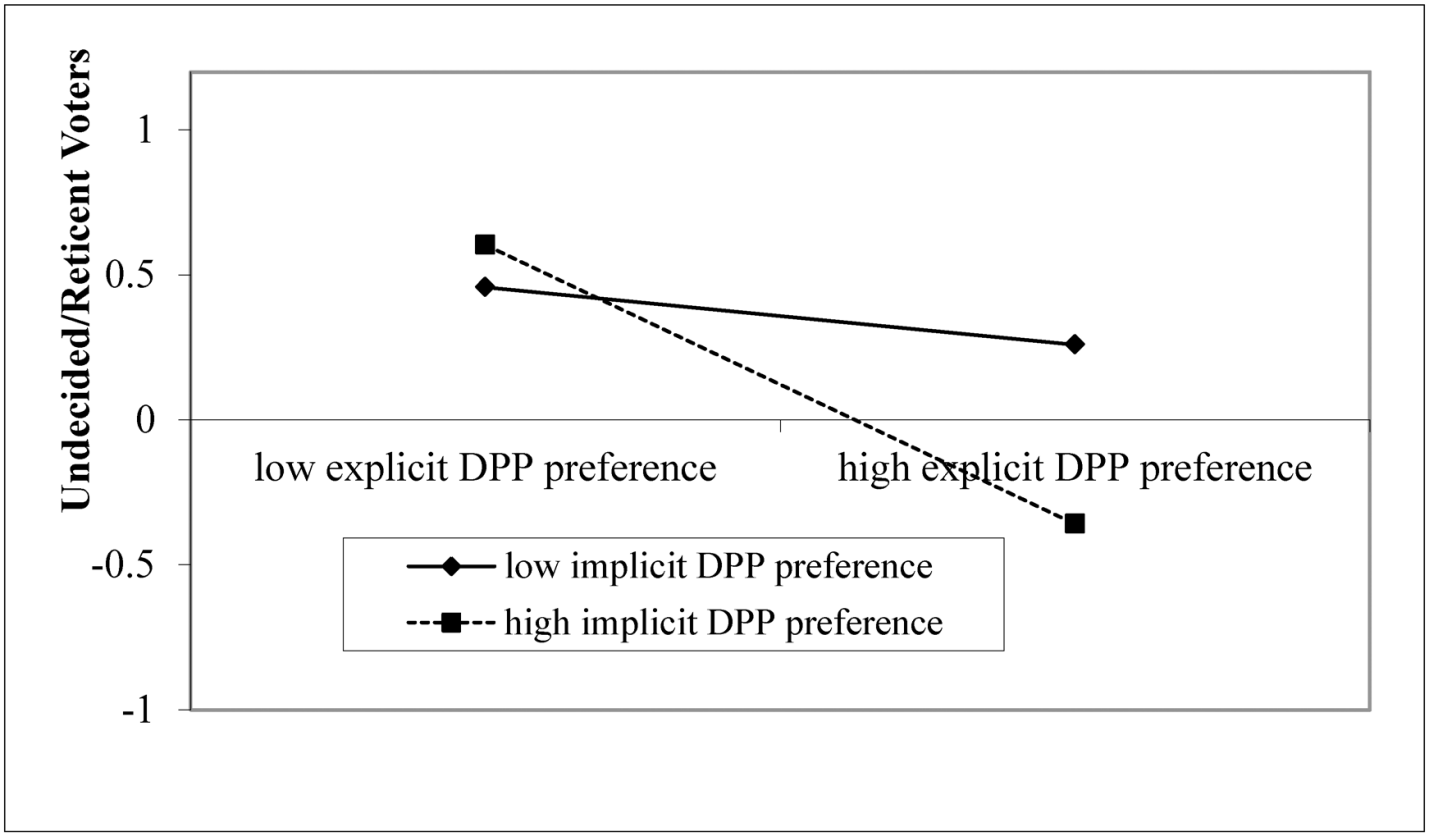
Respondents’ explicit and implicit party preference (DPP over KMT) in predicting undecided/reticent voters before election (coded 1 for undecided/reticent voters; and 0 for decided voters). Solid diamond: low implicit DPP preference. Solid square: high implicit DPP preference.

We tested whether an interaction between explicit and implicit political party preferences may account for reticent or undecided voters, as was reported in Roccato and Zogmaister [23]. Following Roccato and Zogmaister’s conceptualization of reticent and undecided voters, participants who refused or were unable to indicate their intended candidate (coded 1 as reticent or undecided) were separated from those who indicated their intended candidate (coded 0). Indeed, a logistic regression model showed that when participants had lower explicit political party preference for the DPP, *B* = -1.23, *p* = .001, lower implicit political party preference for the DPP, *B* = -0.70, *p* = .037, and when their explicit and implicit political party preferences were inconsistent with one another, *B* = -1.15, *p* = .007, they were less likely to have made a voting decision when questioned prior to the elections (see Fig 4).

## General Discussion

The present study weighs evidence for the comprehensive rationality stance and the bounded rationality stance on human cognition through an examination of voting intention and choices in the 2014 Taipei mayoral elections. In this regional election, we found evidence for comprehensive rationality: Taiwanese voters were largely rational and deliberative, with explicit political party preferences serving as the best predictor of their voting intentions, which in turn served as the best predictor of their voting choices. In addition, voting intention mediated the effects of explicit and implicit political party preferences on voting choices. Our evidence suggests that although implicit attitudes are often treated as a challenge to human rationality [5], this may not be the case [19]. We replicated Roccato and Zogmaister’s findings [23], demonstrating that when participants’ explicit and implicit political preferences did not ally, participants were less likely to express their intended candidates. In other words, taken together with the results from Roccato and Zogmaister [23], the impact of implicit preferences on voting appears to be present across different cultures, which may signal to individuals that they need to process information more cautiously when deciding for whom to vote.

However, we also found evidence to support bounded rationality. The voting choices of our participants were affected by their ethnic identity, even though it was not directly associated with the voting intentions of the participants. There are several plausible explanations for these findings. First, voters may not be aware of the impact of ethnic identity on their decisions. Alternatively, political candidates (e.g., Ko, Lien) may have exerted their efforts in the final days prior to the day of the election to sway the decisions of participants, appealing indirectly to voters’ ethnic identity through their campaigns. Finally, participants may have actively denied the possibility of ethnic identity exerting an influence when reporting on their voting intention, because they found it irrelevant to the mayoral election; but when they actually voted, they were unable to resist the impact of their ethnic identity.

Given that an individual’s social network is often composed of social ingroup members (e.g., those who belong to the same ethnicity), it is perhaps unsurprising that ethnic identity has been found to predict political activities, such as voting preference among Latino populations in the U.S. [28–29] and political involvement among immigrants in Germany [30]. Ethnic identity has often been treated as a group marker in which voters opt for candidates who are members of the same ethnic group. Graves and Lee [28] delineated a theory of ethnic voting, stating that ethnicity may affect voters’ partisanship and candidate evaluation, which in turn affects their voting preferences. That is, voters may favor a political party and candidates endorsed by their ethnic members (e.g., the Latino population typically supports the Democratic Party and its candidates in the U.S.). Voters may evaluate candidates from their ethnic group more favorably than those from different ethnic groups, perceiving these candidates to be more supportive of issues related to their ethnic group (e.g., Latino political candidates may have a more lenient view on immigration).

Additionally, ethnic identity may be viewed as a politicized collective identity [31]. That is, in a society where social power is divided along ethnic lines, members of subordinate ethnic groups may collectively blame an outgroup (or outgroups) for their groups’ predicament. As a result, these group members may engage in political activities to enact change. Our findings suggest that the impact of ethnic identity may be more in line with bounded rationality, as its effects were not predicted by one’s voting intention prior to the election. Thus, it is plausible that individuals vote for a candidate who shares the same ethnic (e.g., Taiwanese) identity without consciously deciding to do so. If individuals vote in blind support of their ethnic ingroup or in opposition of ethnic outgroups, their decisions will not involve substantial systematic processing, potentially undermining the principles and effectiveness of the democratic system.

Lastly, according to the path model, the voting choices of our participants were affected by the perceived voting intention of their significant others, but it was not associated with voting intention after all other variables were simultaneously controlled. The finding suggests that the association between voting intention and perceived voting intention of significant others may be pseudo. Previous research has shown that significant others may affect one’s political behavior, such as voter turnout [32–36], voting consistency [37], and voting choices [35, 38]. The effects of significant others are often conceptualized as a rational act. Significant others may enforce a participation norm so people are more likely to vote [32, 36]. Through discussion, people may gain political information and knowledge from their significant others and may be more likely to vote as a result [34, 36]. Previous research has also shown that when people are in a social network that favors their preferred candidates, they are more likely to vote for them [35–36]. Schmitt-Beck [38] also found that significant others may serve as a filter to reinforce or block media information on the candidates. Thus, it appears that voting is not an individual choice but is embedded in one’s social network. However, in our research, the evidence of perceived significant other’s voting intention may be more in line with bounded rationality, as its effects on voting choices were not mediated by one’s voting intention prior to the election. Our findings suggest that there are ways that significant others may affect us that have not been recognized before. More research investigating the impact of significant others beyond individuals’ awareness will be needed to further understand voters’ behavior.

In summary, although the explicit and implicit political preferences of individuals do exert a powerful influence on their voting intention and behavior, other factors–the ethnic identity and the perceived voting intention of their significant others–play important roles as well. There is a need to explore the influence of social groups and significant others, especially in different types of political systems. Future studies should further examine the degree to which relatively sensitive issues (e.g., ethnic identity) and mainstream cultural values (e.g., the extent of other people’s influence in collectivistic societies and in individualistic societies, such as Taiwan and the U.S. respectively) affect the results of an election. Overall, our findings indicate that although the effectiveness of a democracy should rely on the rational and careful decisions of individual voters, actual democratic societies are imperfect systems, shaped by the issues faced by various social groups within the system and by the mutual impact their citizens have on one another. When looking towards the future of democracy, both in Chinese societies and beyond, we must acknowledge the impact of these factors and incorporate our understanding into the way we educate and motivate the citizens who form the fabric of democratic societies across the world.

## Supporting Information

S1 Appendix The Development of the Political Party Preferences Implicit Association Test (IAT). (DOCX)

S2 Appendix The correlation matrix of the variables in the path model (n = 124)^a^. ^a^missing data were estimated by interpolation method. ^b^standardized scores in the correlations and raw scores in the means and standard deviations. **: *p* < .001, ^+^: *p* < .08.
(DOCX)
