# Supplementary material for: Voting Intention and Choices: Are Voters Always Rational and Deliberative?
Source: PLoS One. 2016 Feb 17;11(2):e0148643. doi: 10.1371/journal.pone.0148643 (PMC4757036; doi:10.1371/journal.pone.0148643)
Supplement: S1 Appendix — (DOCX) [file pone.0148643.s001.docx]

Appendix I: The Development of the Political Party Preferences Implicit Association Test (IAT).

To develop the political party preferences IAT, we first selected pictures representing the two main political parties in Taiwan, the Kuomintang (KMT) and the Democratic People’s Party (DPP). We piloted our stimuli with an independent sample of 31 participants, who viewed KMT- and DPP-related pictures, one at a time. Participants were asked to rate the pictures (using a Likert scale ranging from 0, indicating complete disagreement, to 4, indicating complete agreement) with DPP-related words (e.g., “pan-Green”), KMT-related words (e.g., “pan-Blue”), and control words (adjectives that were politically neutral, e.g., “informative,” “political”). There was adequate agreement amongst pilot participants on the DPP-related words (α = .75), the KMT-related words (α = .65), and the control words (α = .83). Consistent with our expectations, in comparison to the selected eight KMT pictures, participants rated the selected eight DPP pictures to be more pan-Green (M_DPP_ = 2.72 versus M_KMT_ = 1.27, *p* < .001, in a mixed model accounting for the rater effect) and less pan-Blue (M_DPP_ = 0.19 versus M_KMT_ = 2.04, *p* < .001) but did not differ on control items (M_DPP_ = 1.66 versus M_KMT_ = 1.62, *p* = .22).

The IAT was administered in five blocks. In Block 1, respondents were asked to respond to *good* words with a left-key and *bad* words with a right-key (20 trials). In Block 2, participants had to categorize DPP-related and KMT-related images (20 trials). In Block 3, respondents either (a) categorized good words/DPP images with a left-key and bad words/KMT images with a right-key, or (b) good words/KMT images with a left-key and bad words/DPP images with a right-key (60 trials). In Block 4, participants again categorized DPP and KMT images (20 trials); the DPP and KMT categories were presented on sides of the screen opposite from in Block 2 (i.e., if “DPP” had appeared on the right side of the screen in Block 2, it now appeared on the left side in Block 4). Finally, in Block 5, participants categorized the word-image pairings that they had not received in Block 3 (i.e., if participants had categorized good words/DPP images with a left-key in Block 3, here they categorized good words/KMT images with a left-key; 60 trials).
