# Supplementary material for: Voting Intention and Choices: Are Voters Always Rational and Deliberative?
Source: PLoS One. 2016 Feb 17;11(2):e0148643. doi: 10.1371/journal.pone.0148643 (PMC4757036; doi:10.1371/journal.pone.0148643)
Supplement: S2 Appendix — amissing data were estimated by interpolation method. bstandardized scores in the correlations and raw scores in the means and standard deviations. **: p < .001, +: p < .08. (DOCX) [file pone.0148643.s002.docx]

Appendix II: The correlation matrix of the variables in the path model (n = 124)^a^

|  | Explicit political party preferences^b^ | IPPP^b^ | Interaction | TWID | Vote intention | PVI | Vote choice |
| --- | --- | --- | --- | --- | --- | --- | --- |
| Implicit political party preference (IPPP) | .46** |  |  |  |  |  |  |
| Interaction b/w Explicit and Implicit political party preferences | -.32** | -.17+ |  |  |  |  |  |
| Ethnic identity (TWID) | .48** | .37** | -.31** |  |  |  |  |
| Vote intention | .70** | .42** | -.39** | .42** |  |  |  |
| Perceived voting intention of significant others (PVI) | .50** | .36** | -.16+ | .35** | .49** |  |  |
| Vote choices | .49** | .32** | -.34** | .44** | .54** | .42** |  |
| Mean | 1.28 | 0.01 | 0.47 | 2.60 | 5.28 | 2.51 | 0.68 |
| Standard deviation | 4.02 | 0.59 | 0.96 | 0.67 | 5.96 | 7.74 | 0.64 |
| Scale | -10 ~ +10 | N/A | N/A | 1 ~ 3 | -10 ~ 10 | -10 ~ 10 | -1 ~ 1 |

Note. a. missing data were estimated by interpolation method. b. standardized scores in the correlations and raw scores in the means and standard deviations.

**: *p* < .001, +: *p* < .08.
